# Supplementary material for: Complex‐centric proteome profiling by SEC‐SWATH‐MS
Source: Mol Syst Biol. 2019 Jan 14;15(1):e8438. doi: 10.15252/msb.20188438 (PMC6346213; doi:10.15252/msb.20188438)
Supplement: Supplementary file 8 — Dataset EV7 [file MSB-15-e8438-s008.zip › feature_plots_string/Q9BVJ6.pdf]

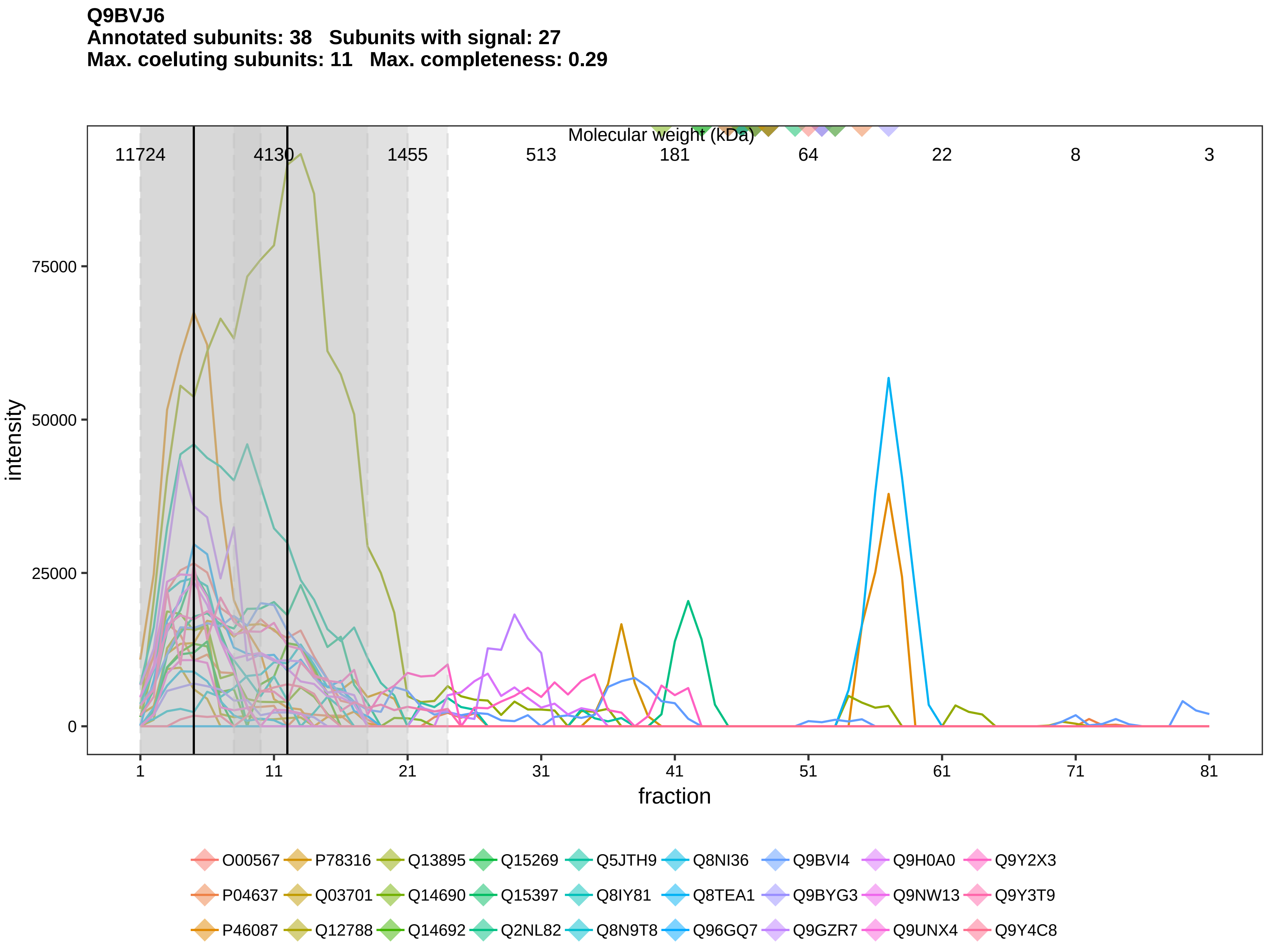
Q9BVJ6
Annotated subunits: 38 Subunits with signal: 27
Max. coeluting subunits: 11 Max. completeness: 0.29
Molecular weight (kDa)
11724
4130
1455
513
181
64
22
8
3
intensity
75000
50000
25000
0
fraction
1
11
21
31
41
51
61
71
81
O00567
P04637
P46087
P78316
Q03701
Q12788
Q13895
Q14690
Q14692
Q15269
Q15397
Q2NL82
Q5JTH9
Q8IY81
Q8N9T8
Q8NI36
Q8TEA1
Q96GQ7
Q9BVI4
Q9BYG3
Q9GZR7
Q9H0A0
Q9NW13
Q9UNX4
Q9Y2X3
Q9Y3T9
Q9Y4C8
